# The Advantages of Hand Pressure in the Introduction and Condensation of Cohesive Gold Fillings

**Published:** 1904-01

**Authors:** Alonzo Milton Nodine


### THE ADVANTAGES OF HAND PRESSURE IN THE INTRODUCTION AND CONDENSATION OF COHESIVE GOLD FILLINGS.

By Alonzo Milton Nodine, D. D. S.

Read before the Second District Dental Society.

It is admitted by most operators that hand pressure possesses not a few advantages, but that this method also has its distinct advantages. The question naturally arises, are the advantages of sufficient value for it to be used in preference to the automatic mallet, in a majority of cavities, and in the major part of nearly all cavities? The attempt will be made to answer this question.

First. Gold may be packed into undercuts with less liability to displacement.

Second. Gold may be lapped over the cervical margin of a cavity, getting closer adaptation, with less liability of fracturing the enamel.

Third. Gold may be more closely adapted to the irregularities of the surface of the tooth structure.

Fourth. Gold may be condensed over cement cappings and linings with less danger of fracturing the cement.

Fifth. Gold may be condensed against frail walls with less danger of fracturing them or checking the enamel.

Sixth. Gold may be packed in cavities not accessible to the automatic mallet, particularly disto-approximal cavities in moars and bicuspids.

Seventh. The danger of displacing a filling by a misdirected blow is much less.

Eighth. The jar upon the tooth and patient is not nearly as severe.

Ninth. Hand pressure can produce a filling nearly as rense as one made by mallet force; often one equally as dense and again others that are even denser; this depends upon the operator and the form of gold.

Tenth. The gold remaining more cohesive, there is less flaking of gold.

The statement has been made by several authorities that cohesive gold fillings inserted by hand pressure in holes in a steel plate are not as dense as those made by mallet force.

Here are two fillings, one inserted by hand pressure, the other by the automatic mallet, using the same form of gold. The filling made by the mallet was composed of smaller pieces of gold. (Exhibits fillings.)

These advantages result from the fact that in condensing the gold there is no sudden impact to spring the gold, no penetrating blow to pierce the gold and injure the margins; instead there is a steady pushing and rocking motion that works the gold into the inequalities.rather.than driving it in.

In a paper written by Dr. Black and published in the September *C sm s* of 1895, are the results of a series of experimental fillings, made by several operators *us ng different methods.* . .

There were but two operators that used cohesive gold and also used mallet force in one cavity and hand pressure in the other.

Dr. S. inserted three fillings, one using hand pressure getting a specific gravity of 12.

One using the automatic mallet (each piece of gold receiving twenty-five blows) specific gravity of 12.5.

One using the automatic mallet (each piece of gold receiving fifty blows) specific gravity of 14.0.

Dr. H., using hand pressure, getting specific gravity of 16.9; using hand mallet (50 blows upon each piece of gold) specific gravity of 17.4.

Dr Black read a paper January 21, 1896, before the New York Odontological Society, in which were given the results of other experimental fillings.

There was but one operator using cohesive gold, who also used hand pressure in one cavity and mallet force in the other.

Dr. J. F. P. Hudson produced with hand pressure, using Watts's gold, a filling having a specific gravity of 17.76 and one with the automatic mallet getting a specific gravity of 18.05.

Dr. Johnson, in his work on "Operative Dentistry," says of fillings inserted by hand pressure: "While they are not as dense, they usually succeed in saving teeth. They do this by reason of good adaptation to the cavity walls." Further he says, "It is not the hardest worked gold filling that is always the best, but the one most closely filling the walls of the cavity and which is the most uniformly packed."

Dr. Ottolengui in "Methods of Filling

Teeth" says, "The greatest good gained by hand pressure is that the gold remains more cohesive under hand pressure than in connection with any other. The more gradual the pressure exerted upon gold foil in condensing it the less it loses its quality of cohesiveness, and vice versa, the more sudden or rapid the blow of the hammer, the less cohesion will be exhibited."

Dr. Louis Jack in the "American System of Dentistry" in mentioning the three steps in condensing gold by hand pressure says, "The third movement is one of leverage, which, being one of the powerful mechanical forces, is more efficient in effecting consolidation than the greatest amount of direct force applicable could be.

It has been shown that great force is not needed to bring about the cohesion of gold when it has been correctly prepared.

The gold is laid upon the part with some direct pressure, which is immediately followed by a tilting or rocking movement of the instrument. This produces leverage in which one corner of the instrument is the fulcrum and the other coming down with energy, overcomes the irregularities of the suface and also produces contact and therefore union.

